# Antimicrobial resistance pattern of Gram-negative bacilli isolated from patients at ICUs of Army hospitals in Iran

**Published:** 2011-03

**Authors:** M Mohammadi-mehr, MM Feizabadi

**Affiliations:** 1 Department of Microbiology, School of Medicine, Army University of Medical Sciences; 2 Department of Microbiology, School of Medicine, Tehran University of Medical Sciences, Tehran, Iran

**Keywords:** Gram-negative, ICU, Antimicrobial resistance, Antibiotic

## Abstract

**Background and Objectives:**

Patients at intensive-care-unit (ICU) are at risk of acquiring nosocomial infections which contributes to higher rates. Approximately 25% of all hospital infections and 90% of outbreaks occur in ICUs. Multi- resistant gram-negative rods are important pathogens in ICUs, causing high rate of mortality. The purpose of this study was to investigate the antimicrobial resistance patterns among common Gram-negative bacilli isolated from patients with nosocomial infection at Army Hospitals.

**Materials and Methods:**

A total of 187 isolates of Gram-negative bacilli were isolated from 904 patients at ICUs of three Army hospitals in Iran during May 2007 to May 2008. All isolates were examined for antimicrobial resistance using disc diffusion method.

**Results:**

The most frequent pathogens were *E. coli* (32.08%) followed by *K*. *pneumoniae* (31%), *P. aeruginosa* (12.8%) and *Acinetobacter* spp. (9.1%). High rate of resistance to third generation cephalosporines was observed among isolates of *E. coli* and *K. pneumoniae*. Production of extended spectrum beta lactamases (ESBLs) was found in 46.6% of isolates of both organisms, but in 38% of all Gram negative bacteria.

**Conclusion:**

The prevalence of ESBL producing strains at three Army Hospitals is considerable (38%). However, resistance to imipenem has emerged in these hospitals. Furthermore, studies are required to clarify the situation with multi-drug resistant organisms including the Gram positive bacteria at Army hospitals.

## INTRODUCTION

A considerable number of critically ill patients, in particular those staying in ICUs, acquire different infections following hospitalization (1–4). Many factors affect the risk of nosocomial infections including underlying disease, severity of illness, length of ICU stay, and usage of invasive devices and procedures. These infections have been reported to affect approximately 2 million hospitalized patients in the US annually and have imposed 57.6 billion dollars in expense to the US health system in 2000 (5, 6). The beds at ICUs consisted of 5 to 10% of all hospital beds but they account for 10–25% of health-care costs generated (7). The patient in the ICU has a 5- to7-fold higher risk of nosocomial infection compared with the other patients. This is a consequen-ce of impaired defense mechanism, applying invasive methods and monitoring devices, exposure to broad-spectrum antibiotics, and the colonization of resistant microorganisms. The frequent use of broad-spectrum antibiotics results in colonization with resistant Gram-negative bacteria and consequently in serious infec-tions (8, 9). ICUs accommodate the most seriously ill patients in a relatively confined environment. Increased duration of stay, increased number of indwelling devices and prolonged or inappropriate use of antibiotics on the ICU-leading to selection of multi-resistant ‘super- bugs’-are all associated with significantly increased mortality (10–12). The highest rates of nosocomial infections are observed in ICUs, which are also the units in which the most severely ill patients are treated and in which the highest mortality rates are observed (13). The purpose of this study was to investigate the antimicrobial resistance patterns among gram- negative bacteria isolated from patients who were admitted to ICUs and acquired infection at 3 army hospitals in Tehran.

## MATERIAL AND METHODS

This is a cross-sectional study carried out in 3 Army hospitals (A, B, C) in Tehran, Iran, during May 2007 to May 2008.

**patients and Bacterial strains**. All bacterial isolates recovered from patients of ICUs were identified based on their characteristic appearance on the media and the patterns of biochemical reactions using conventional bacteriological methods (14). Patients were included in the study if they showed no sign of infection at the time of admission and were not in the incubation period of any infection. They were diagnosed to have nosocomial infections within 48 to 72 h following their admission as defined by the Centers for Disease Control (15). The specimen sources included blood, urine, cerebrospinal fluid, respiratory tract (collected during bronchoscopy or endotracheal suction), tip of central venous catheters, bedsores, and surgical incision sites.

**Susceptibility testing**. The susceptibility of the isolated gram negative bacteria were tested against imipenem (10 µg), nitrofurantoin (100 µg), ampicillin (10 µg), trimethoprim/sulfomethoxazole (1.25/1.23 µg), ciprofloxacin (30 µg), cefotaxim (30 µg), ceftazidime (30 µg), cefepime (30 µg), piperacillin (100 µg), amikacin (30 µg), ceftriaxone (30 µg), piperacillin- tazobactam (100/10 µg), gentamicin (30 µg) using the Kirby-Bauer disc-diffusion technique as described by the NCCLS (16). Quality control was assured by concurrent testing with the American Type Culture Collection (ATCC) strains including *E. coli* ATCC 25921, *P. aeruginosa* ATCC 27852 and *Staphylococ- cus aureus* ATCC25923.

The combined disks (10 µg of clavulanic acid plus 30 µg of ceftazidime/cefotaxime) in parallel with these cephalosporines were used for detection of extended spectrum beta-lactamases (ESBLs) (17).

**Statistical analysis**. Statistical analysis was performed using SPSS version 16. All tests were two- tailed and p<0.05 was considered significant.

## RESULTS

Of 904 patients who stayed at ICUs, 187 (20.8%) were infected with Gram negative bacilli during May 2007-May 2008 and these belonged to Hospitals: A (n=70, 37.43%), B (n=48, 25.66%) and C (n=69, 36.89%). Other organisms causing nosocomial infec- tions in these hospitals were *Candida* spp., (n=36) and gram positive bacteria (n=50).

Among the studied patients, 56.1% (105 cases) were male and 43.9% (82 cases) were female. The mean age of the patients was 64.46 years (range 16–95, SD±19.8). The mean hospitalization for patients with nosocomial infections was 17.27 days. The primary reasons for hospitalization were malignancies, infections, fracture, operation, kidney dysfunction, poisoning, convulsions and cardiac diseases. Patients with heart disease were the largest group who got nosocomial infections in this study. The underlying disease such as diabetes, cardiac and renal dysfunction were found in 121 (64.7%) of patients. Patients who had undergone implant utilization made 73.8% (n=138) of population (with a mean of 11.36 days implant usage). Of 187 patients in this study, 57 (30.5%) had history of operation.

Statistical analysis found correlation between usage of antibiotics and occurrence of nosocomial infection. There was not significant difference between the genders and nosocomial infection (P<0.05). The mean duration of stay at ICUs was 10.34 days (SD±11.21). The mean duration for hospital stay for patients who got nosocomial infection was 17.27 days (17.57).

The most frequent nosocomial infection was urinary tract infection (UTI) (106 patients, 56.7%) followed by pneumonia (73 patients, 39%), primary septicemia (5 patients, 2.7%), surgical site infection (3 patients, 1.6%). The frequency of different gram negative bacteria caused UTI, pneumonia, septicemia and surgical site infections in this study is shown in Table 1.

**Table 1:** Frequency of Gram-negative bacteria isolated from 187 patients with different nosocomial infections at Army hospitals.

Microorganisms	**Number of patients (%) infected with nosocomial Infections**				Total (%)

	UTI	pneumonia	primary septicemia	Surgical site infection	
*Escherichia coli*	54 (50.94%)	5 (6.84%)	0 (0)	1 (33.33%)	60 (32.08)
*Klebsiella Pneumoniae*	11 (10.37%)	45 (61.64%)	1 (25%)	1 (33.33%)	58 (31.1)
*Pseudomonas aeruginosa*	12 (11.32%)	12 (16.43%)	0 (0)	0 (0)	24 (12.8)
*Acinetobacter bumannii*	10 (9.4%)	5 (6.84%)	2 (40%)	0 (0)	17 (9.1)
*Enterobacter aerogenes*	6 (5.6%)	4 (5.47%)	2 (40%)	0 (0)	12 (6.4)
*Proteus mirabilis*	7 (6.6%)	0 (0)	0 (0)	1 (33.33%)	8 (4.3)
*Citrobacter*	4 (3.77%)	1 (1.36%)	0 (0)	0 (0)	5 (2.7)
*Seratia*	2 (1.88%)	0 (0)	0 (0)	0 (0)	2 (1.1)
*Alcaligenes*	0 (0)	1 (1.36%)	0 (0)	0 (0)	1 (0.5)

High rate of resistance to third generation cepholos- porins was observed among isolates of *E. coli* and *K. pneumoniae*. Production of ESBLs was found in 46.6% of isolates of both organisms, but in 38% of Gram negative bacteria. Bacteria other than *E. coli* and *Kebsiella pneumoniae* also showed resistance to beta lactam containing antibiotics (Table 2). For example, all isolates of *Acinetobacter baumannii* were resistant to ampicillin, cefotaxime and ceftriaxone (100%). Other antibiotics including ciprofloxacin and aminoglyosides poorly responded *in vitro* (Table 2).

**Table 2:** Proportion of resistance to different antibiotics among gram negative bacteria isolated from patients at 3 army hospitals.

Antibiotics	* E. coli n (%) *	* K. pneumoniae *	* P. aeruginosa *	* A. bumannii *	* E. aerogenes *	* P. mirabilis *	* C. ferundii *	* Seratia *	* Alcaligenes *
Imipenem	1 (1.6)	2 (3.4)	4 (16.66)	7 (41.2)	0 (0.0)	1 (12.5)	0 (0.0)	0 (0.0)	0 (0.0)
Nitrofurantoin	10 (16.66)	33 (56.9)	24 (100)	16 (94.1)	4 (33.3)	6 (75.0)	3 (60.0)	0 (0.0)	0 (0.0)
Ampicillin	53 (88.33)	57 (98.3)	22 (91.66)	17 (100)	12 (100)	8 (100)	5 (100)	2 (100)	1 (100)
Trimethoprim/ Sulphamethoxazole	35 (58.33)	37 (63.8)	21 (87.5)	14 (82.4)	5 (41.7)	6 (75.0)	4 (80.0)	1(50.0)	1 (100)
Ciprofloxacin	36 (60.0)	37 (63.8)	6 (25.0)	9 (52.9)	5 (41.7)	1 (12.5)	3 (60.0)	1 (50.0)	1 (100)
Cefotaxime	36 (60.0)	48 (82.8)	16 (66.66)	17 (100)	7 (58.3)	5 (62.5)	3 (60.0)	1 (50.0)	1 (100)
Ceftazidime	37 (61.66)	48 (82.8)	10 (41.66)	16 (94.1)	7 (58.3)	2 (25.0)	2 (40.0)	1 (50.0)	1 (100)
Cefepime	41 (68.33)	49 (84.5)	14 (58.33)	16 (94.1)	7 (58.3)	5 (62.5)	2 (40.0)	1 (50.0)	1 (100)
Piperacillin	45 (75.0)	50 (86.2)	10 (41.66)	15 (88.2)	6 (50.0)	5 (62.5)	5 (100)	1 (50.0)	1 (100)
Amikacin	7 (11.66)	34 (58.6)	16 (66.66)	13 (76.5)	5 (41.7)	2 (25.0)	1 (20.0)	1 (50.0)	1 (100)
Ceftriaxone	36 (60.0)	45 (77.6)	14 (58.33)	17 (100)	6 (50.0)	3 (37.5)	2 (40.0)	1 (50.0)	1 (100)
Piperacillin-tazobactam	20 (33.33)	35 (60.3)	5 (20.83)	6 (35.3)	3 (25.0)	2 (25.0)	1 (20.0)	1 (50.0)	1 (50.0)
Gentamicin	19 (31.66)	29 (50.0)	6 (25.0)	13 (76.5)	6 (50.0)	4 (50.0)	2 (40.0)	1 (50.0)	1 (100)

## DISCUSSION

Antimicrobial resistance is an increasingly emerging problem worldwide, especially in ICUs. Identifying the resistance pattern of microorganisms in every hospital is the key to success in the appropriate treatment of patients. The incidence of nosocomial infections with gram negative bacteria in the studied hospitals was 21%.

*E. coli* and *K. pneumoniae* were the most common organisms involved in UTI and respiratory tract infections respectively. In our study, the most frequent nosocomial infection was urinary tract infection (56.7%) followed by pneumonia (39%), primary septicemia (2.7%), surgical site infection (1.6%). This is different from previous report from a teaching hospital in Tehran where primary septicemia (28.4) followed by pneumoniae (25.8%) had been found as the most frequent nosocomial infection (18). Pneumonia has also been reported as the most frequent nosocomial infection in Turkey (938.8%), India (29.5%) and Oman (65%) (8, 19, 20). *K. pneumoniae* and *E. coli* were the most frequent agents from respiratory specimens and urinary tracts. *E. coli* has been reported as the most common microorganism isolated from urinary tracts in Oman (19).

Beta-lactams are the most widely used antibiotics all over the world, and resistance to this antibiotic has resulted in a major clinical crisis (21). In Iran, cepholosporins are widely used due to their low rate of side effects. This might be associated with the increased risk of antimicrobial resistance.

The findings of the present study are indicative of high resistance rates in most microorganisms. In the current study, the most frequency of resistance to antimicrobial agents was 50% to 100% for ceftriaxone, 50% to 94.1% for ceftazidime, 52.9% to 63.8% for ciprofloxacin, 58.3% to 84.5% for cefepime, 56.9% to 100% for nitrofurantoin, 88.33% to 100% for ampicillin, 58.335 to 87.5% for trimethoprim/ sulphamethoxazole, 58.3% to 100% for cefotaxime, 60% for piperacillin- tazobactam and 50% for gentamicin.

The rate of resistance to ceftriaxone found in this study is lower than the previous report (22, 23).

The resistance frequency to imipenem was 1.6% for *E. coli*, and 3.4% for *K. pnumoniae* and 16.66% for *P. aeruginosa*, 41.2% for *A. bumannii* and 12.5% for *P. mirabilis* (Table 2). This is different from previous studies since isolates of *Klebsiella spp*. and *E. coli* had been fully susceptible to imipenem. (19, 24). Resistance to ceftazidime was 82.2% in our study that is higher than similar studies (25–27).

In this study, the frequency of resistance to at least 3 antibiotics was 94.1% in case of *A. baumannii*. And 82.8% in case of *K. pneumoniae*. In a study conducted in Turkey, multidrug resistance was reported to be 45.4% in case of *Acinetobacter spp*. and 37.7% in case of *P. aeruginosa* (28).

The prevalence of ESBLs among the isolates of *E. coli* and *K. pneumoniae* (46.6%) in this study is close to the prevalence of *bla*_CTX-M’_ one of the most frequent gene encoding ESBL, at one of the general hospital in Tehran (29). Other gram negative bacteria also showed resistance to beta lactam containing antibiotics (Table 2). For example, all isolates of *A. baumannii* were resistant to ampicillin, cefotaxime and ceftriaxone (100%). Other antibiotics including ciprofloxacin and aminoglyosides poorly responded *in vitro* (Table 2).

Considering the increasing antimicrobial resistance rate in hospitals, a committee for rational drug administration is needed to collaborate with infection control committees. Most ICU-associated infections can be prevented and controlled by applying the principle procedure and these include hand-washing, timely use and cessation of antibiotic therapy, timely change or removal of indwelling ‘lines’, early extubation and physiotherapy. The clinical specimens should be subjected to bacterial culture and antibiotic susceptibility testing prior to antibiotic therapy to determine the appropriate drug.
